# Effects of Changes in Water Intake on Mood of High and Low Drinkers

**DOI:** 10.1371/journal.pone.0094754

**Published:** 2014-04-11

**Authors:** Nathalie Pross, Agnès Demazières, Nicolas Girard, Romain Barnouin, Déborah Metzger, Alexis Klein, Erica Perrier, Isabelle Guelinckx

**Affiliations:** 1 Forenap, Rouffach, France; 2 Biotrial Neuroscience, Didenheim, France; 3 Danone Research, Palaiseau, France; University of California, San Francisco, United States of America

## Abstract

**Objective:**

To evaluate the effects of a change in water intake on mood and sensation in 22 habitual high-volume (HIGH; 2-4 L/d) and 30 low-volume (LOW; <1.2 L/d) drinkers who were asked to respectively decrease and increase their daily water intake.

**Method:**

During baseline HIGH consumed 2.5 L and LOW 1 L of water/day. During 3 controlled intervention days HIGH's water intake was restricted to 1 L/day whereas LOW's was increased to 2.5 L water/day. Several mood scales (Bond & Lader Visual Analog Scale (VAS), Profile of Mood States, Karolinska Sleepiness Scale, Thirst & Emotional VAS) were administered at different time points during the study. ANOVA including intervention, time point and intervention by time point as fixed effects on mean values (i.e.; baseline data *vs.* mean of 3 intervention days) for each mood scale was performed.

**Results:**

At baseline HIGH and LOW were comparable in mood state, except for thirst scores (estimate = 17.16, p<0.001) and POMS depression-dejection scores (estimate = 0.55, p<0.05) which were both higher in the HIGH *vs.* LOW. In HIGH the restricted water intake resulted in a significant increase in thirst (p<0.001) and a decrease in contentedness (p<0.05), calmness (p<0.01), positive emotions (p<0.05) and vigor/activity (p<0.001). In LOW, increased water consumption resulted in a significant decrease in fatigue/inertia (p<0.001), confusion/bewilderment (p = 0.05) and thirst (p<0.001) and a trend to lower sleepiness (p = 0.07) compared to baseline.

**Conclusion:**

Increasing water intake has beneficial effects in LOW, especially sleep/wake feelings, whereas decreasing water intake has detrimental effects on HIGH's mood. These deleterious effects in HIGH were observed in some sleep/wake moods as well as calmness, satisfaction and positive emotions.

## Introduction

There is a growing body of literature showing that fluid deprivation negatively impacts several mood areas [1]. However most of these studies were performed in extreme conditions (e.g.: heat, intense physical exercise, high level of dehydration) and/or on specific populations like soldiers or athletes [2]–[5]. Consequently, extrapolation of these study results to a healthy population experiencing only mild changes in water balance during normal activities of daily living is difficult. Moreover, in the few studies targeting the general population, the effects of mild dehydration on mood and sensation have rarely been explored. When studied, the most consistent effects of mild dehydration on mood are related to sleep/wake mood impairments (i.e.; increased fatigue and decreased vigor/activity) and an increase in complaints of headache, thirst, sleepiness and concentration difficulties [6]–[11].

Studies examining the effects of increased water consumption on mood and physiological sensations (e.g. thirst and headache) are even rarer. To the best of our knowledge, only three studies on this topic have been performed in healthy adults [12]–[14]. Rogers and colleagues [12] showed in adults an immediate positive effect of water consumption on mood (only alertness was tested) and a dose dependent reduction in thirst. However, this beneficial effect of water on alertness was only significant two minutes after drinking, and no longer observed 50 min after consumption. The positive effect of water ingestion on self-rated alertness has subsequently been both confirmed [13] and refuted [14]. Among four similar studies conducted in young children, inconsistent effects of water consumption have been reported for memory, attention, and mood [15]–[18]. Specific to mood assessment, Edmonds et al. [18] reported that up to 500 mL water intake had beneficial effects on happiness ratings, compared to a control condition. However, in a second study that offered only 250 mL of water, no difference in happiness was reported between both conditions. Thus, the existing literature on the effect of water consumption on mood is inconsistent. Furthermore, previous research has evaluated immediate (within one hour) effects of water consumption; changes in habitual daily intake volume have not been assessed.

The aim of the current study was to evaluate the effect of a change in water intake on several mood states and physiological sensations in high (HIGH; habitual fluid intake ≥2 L/d) and low (LOW; habitual fluid intake <1.2 L/d) drinkers who were asked to respectively decrease and increase their daily water intake. Based on the limited literature, it was hypothesized that in HIGH, a water decrease would negatively impact sleep/wake related moods. For LOW it was hypothesized that increased water intake would positively affect alertness and happiness ratings, as previously demonstrated in adults and young children.

## Materials and Methods

### Ethics Statement

This study was conducted at a single center in accordance with the ethical principles stated in the revised version of the Declaration of Helsinki and after approval on October 12^th^ 2010 by an Independent Ethics Committee, Comité de Protection des Personnes Est IV, located in Strasbourg. The protocol was also approved by an institutional review board of the French Drug Agency, Agence Nationale de Sécurité du Médicament de Santé, on September 23rd 2010. All participants provided written informed consent.

### Study design and procedures

After an eligibility check and before inclusion in the study, potential subjects were asked to report their habitual fluid intake at home in an electronic diary (Neometis-24WQ-Waters questionnaire) for three consecutive days. Among the 87 subjects enrolled, 35 were not included for the following reasons: habitual daily fluid consumption not consistent with inclusion criteria (N = 15), consent withdrawal (N = 9), other reasons (e.g.; positive urine test, poor venous system, N = 11). Eligible subjects whose total daily fluid consumption was consistent with the thresholds for low (LOW, <1.2 L/day, N = 30) or high fluid intake (HIGH, ≥2 L/day, N = 22) were included in the study. These cut-offs for fluid intake were based on previous research indicating that one third of the French population consumed less than 1.2 L of fluid per day and that one third consumed more than 2 L per day [19], [20]. By using these cut-offs for fluid intake, we aimed to select a study population representative of at least two thirds of the French population. Other inclusion criteria were the ability to provide written informed consent, a willingness to remain at the study center for the duration of the inpatient study period and refrain from intensive physical exercise throughout the study. For all female subjects, an additional inclusion criterion was the use of a monophasic oral contraceptive. Exclusion criteria included smoking, habitual high caffeine (>250 mg/day) or alcohol (>20 g/day) consumption, or the use of any over-the-counter or prescription medications. In total fifty-two young healthy adults (11 men and 41 women) participated in this non-randomized and non-blinded study.

**Figure 1:**
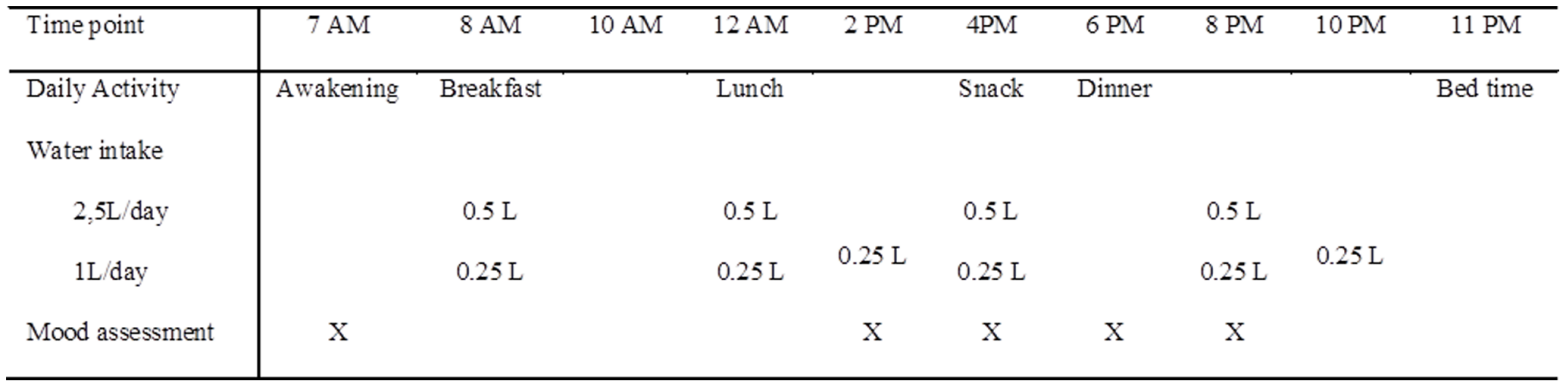
Investigational schedule. Note: X corresponds to the mood and sensations assessments (i.e.; Thirst VAS, KSS, eVAS, POMS, Bond and Lader VAS).

The inpatient study period lasted 6 days (from the afternoon of Day 0 to the morning of Day 6) for both groups. All meals, sleep and waking hours were standardized. To minimize water lost to sweat, participants were permitted only sedentary activities such as reading, playing cards, or watching television. A drinking program was defined for each group (Figure 1) to standardize the volume of water consumed and to control the timing of intake. During the baseline period (Day 1 and Day 2), HIGH were provided with 2.5 L of natural mineral water (Volvic, France) per day, whereas LOW were provided with 1 L per day. During the intervention period (Day 3, 4 and 5), HIGH reduced their water intake to 1 L per day whereas LOW increased their intake to 2.5 L per day. No additional beverages were permitted during the inpatient period. Water provided with a meal was always divided into two equal parts, given immediately before and after each meal. A study nurse verified compliance with the water intake protocol. In addition to the mood variables, physiological measures of hydration were recorded at multiple time-points daily. The physiological responses to this modified drinking program are presented elsewhere [21].

### Subjective mood and physiological sensations

Since circadian variations in mood have previously been reported [22], [23], mood and sensation assessments were repeated several times during the day (07:00, 14:00, 16:00, 18:00 and 20:00). The following assessments were administered in both groups at baseline (Day 1) and during the intervention days (Day 3, Day 4 and Day 5) in the following order:

- The ***thirst Visual Analogue Scales (tVAS)*** [24] is a self-rated 100-mm-line designed to measure the sensation of thirst. The higher the score, the higher the corresponding symptom.
- The ***Karolinska Sleepiness Scale (KSS)*** [25] is a 9-point self-rated scale that quantifies the subjective sensation of sleepiness, with higher scores indicating a higher level of sleepiness.
- The ***emotional Visual Analogue Scale (eVAS)***, adapted from [26] has been used to subjectively assess emotional stability. The e-VAS is a self-rated 100-mm-line with a sad smiley face on one end, and a happy smiley face on the other end. The e-VAS score varies between 100 (very happy) and 0 (very sad).
- The ***Profile Of Mood States*** [27] is a 5-point self-administered scale that assesses various mood states. The POMS consists of 65 items which measures 6 different factors: Tension-Anxiety, Depression-Dejection, Anger-Hostility, Fatigue-Inertia, Vigor-Activity, and Confusion-Bewilderment. The greater the score, the greater the corresponding mood state.
- The ***Bond and Lader Visual Analogue Scales*** [28] consist of 16 bipolar, 100 mm horizontal lines with two opposing adjectives at either end. The subject marks a vertical bar on the line corresponding to how he is feeling right now. The Bond and Lader VAS is analyzed to derive three factor sub scores: alertness, contentedness (well-being) and calmness. The scores are expressed in millimeters. An increase in the score signifies a decrease in the intensity of the corresponding feeling.

### Statistical analyses

All statistical analyses were performed with SAS version 9.1.3 (Cary, NC) with data from all subjects who completed the study. To compare LOW and HIGH groups at baseline, an ANOVA including group, time point and group by time point as fixed effects for each parameter was performed. Males were not included in the baseline comparisons since they were only represented in the LOW group.

To assess the effects of water intake switch in LOW and HIGH (i.e.; intervention effect) and to include circadian mood variations, an ANOVA including intervention, time point and intervention by time point as fixed effects on mean values (i.e.; Day 1 data *vs* mean of Day 3-4-5, but keeping the various time points separated) was performed separately on the LOW and HIGH groups. Analyses were also performed on each day separately, but the results are not presented here as the results were similar and did not provide further insight into the effects of a water intake change on mood and physiological sensation.

Results are presented as estimates (E), 95% confidence intervals (95% CI) and p-values (statistical level of significance at p<0.05, two-tailed). Means and standard deviations for the different mood scales are provided in the tables.

## Results

### Subjects

All 52 randomized subjects completed the study protocol and were included in the analyses. Based on fluid consumption at screening, 30 LOW and 22 HIGH were identified. Participant characteristics and habitual daily fluid intake volume, as reported during the screening period, are provided in Table 1. At baseline, 24 h urine osmolality in HIGH was 333±58 mOsm•kg^−1^; this increased to 720±146 mOsm•kg^−1^ response to a decrease in water intake (p<0.001). 24 h urine osmolality in LOW was 841±206 mOsm•kg^−1^ at baseline and 392±92 mOsm•kg^−1^ after increasing daily water intake (p<0.001). Detailed physiological data on additional hydration biomarkers have been reported elsewhere [21].

**Table 1:** Summary of demographic characteristics and fluid consumption habits of study population.

		All	Low drinker	High drinker
Number	n	52	30	22
**Age**, years	Mean ± SD	24.8±3.1	24.9±2.9	24.5±0.7
	Min – Max	20–30	20–30	20–30
**Sex**, n (%)	Male	11 (21.2)	11 (36.7)	-
	Female	41 (78.8)	19 (63.3)	22 (100.0)
**BMI**, kg/m^2^	Mean ± SD	22.32±1.61	22.38±1.59	22.25±1.67
	Min – Max	20–25.1	20–25.1	20–24.9
**Total fluid intake**	Mean ± SD	1.54±1.07	0.71±0.28	2.66±0.65
**during screening**, L	Min – Max	0.12–5.33	0.12–1.19	2.00–5.33

### Group comparisons at baseline

**Figure 2:**
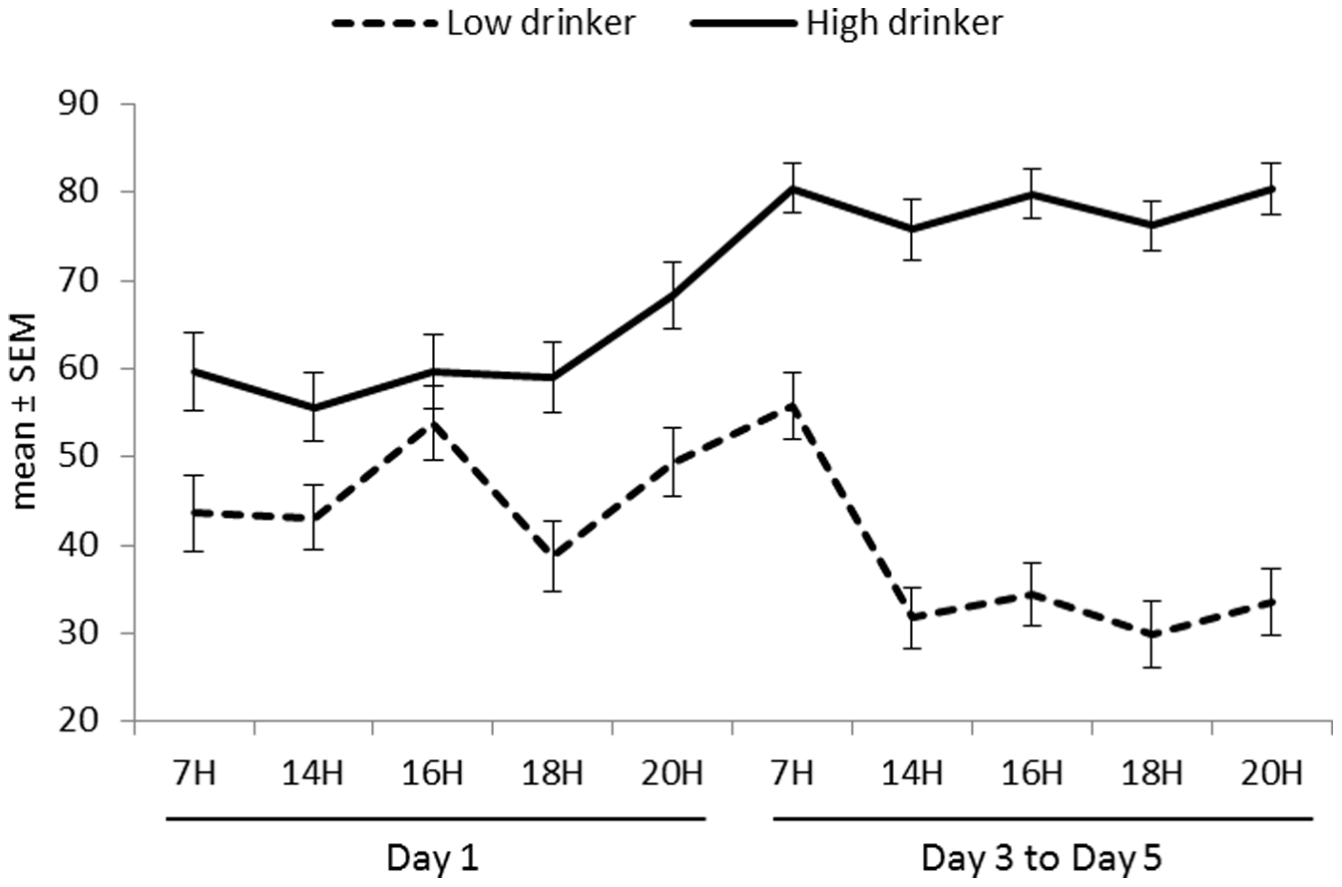
Plots of thirst scores (mean ± SEM) over time according to groups and days.

At baseline, HIGH and LOW were in comparable mood state, except for the thirst scores and the POMS depression-dejection scores. HIGH reported higher thirst feelings compared to LOW (group effect p<0.001, estimate  = 17.16, 95% CI = [11.43; 22.88]) (Figure 2). The depression scores in HIGH were higher than those in LOW (group effect p<0.05, estimate  = 0.55, 95% CI = [0.6; 1.04]).

### Effects of reduced water intake in HIGH drinkers

In HIGH, the Bond and Lader VAS scores revealed significant decreases in contentedness and calmness (p<0.05 and p<0.01, respectively; Table 2). On the eVAS there was a decrease in positive emotions (p<0.05). On the POMS, there were significant intervention effects for the fatigue-inertia (p<0.05) and vigor-activity (p<0.001) scales, indicating the somewhat contradictory finding that HIGH were both less vigorous and less fatigued after the water intake switch. Thirst significantly increased after the reduction in water intake (intervention effect p<0.001).

**Table 2:** Mean values and standard deviation for the different mood scales according to time point & study period (baseline vs mean of Day 3-4-5) for the high drinkers group consuming 2.5 L/day at baseline and 1 L/day during intervention days.

Outcome parameters	Time point					p value (for main intervention effect)
	07:00	14:00	16:00	18:00	20:00	
**POMS**						
**Tension**						
Day 1	−0.7±3.1	−2.3±1.5	−2.1±1.2	−1.7±1.6	−1.9±1.6	ns
Day 3-4-5	−0.9±1.5	−1.8±1.2	−1.3±1.5	−1.0±2.0	−1.7±1.6	
**Depression**						
Day 1	1.2±2.6	1.3±1.9	0.9±2.2	0.5±1.1	1.1±2.5	ns
Day 3-4-5	0.7±1.5	0.8±1.4	1.0±2.3	1.0±2.1	0.9±1.8	
**Anger**						
Day 1	1.0±2.2	0.6±0.9	0.5±1.0	0.2±0.6	0.2±0.8	ns
Day 3-4-5	1.0±1.2	0.4±0.9	0.6±1.4	0.5±1.3	0.6±1.1	
**Fatigue**						
Day 1	5.1±2.3	3.6±2.9	2.5±2.1	2.4±2.1	3.7±3.8	p<0.05
Day 3-4-5	3.9±2.5	2.5±2.0	2.7±2.2	2.1±1.7	2.2±1.8	
**Confusion**						
Day 1	−0.6±2.1	−1.2±1.9	−1.4±1.5	−1.3±1.6	−1.0±3.0	ns
Day 3-4-5	−0.5±1.6	−1.1±1.3	−1.1±1.6	−0.9±1.3	−1.1±1.1	
**Vigor**						
Day 1	10.3±4.8	12.8±4.9	11.2±4.4	11.3±5.4	8.6±4.1	p<0.001
Day 3-4-5	5.7 ±3.4	8.4±4.3	8.4±4.1	8.1±3.8	7.5±4.2	
**KSS**						
Day 1	3.6±1.1	2.8±1.3	2.7±1.1	2.8±1.6	3.2±1.2	ns
Day 3-4-5	4.2±1.0	2.6±1.2	2.6±1.0	2.5±0.9	2.7±0.9	
**eVAS**						
Day 1	67.4±16.8	76.3±14.9	74.3±12.2	71.8±13.4	69.5±8.3	p<0.05
Day 3-4-5	59.8±12.0	69.1±13.2	72.0±10.7	69.0±12.1	70.9±11.3	
**B&L VAS**						
**Alertness**						
Day 1	36.4±10.0	30.9±9.8	30.5±9.7	31.6±8.9	37.1±13.4	ns
Day 3-4-5	41.6±12.8	32.9±12.3	33.0±13.6	32.4±11.8	33.0±11.9	
**Calmness**						
Day 1	25.7±12.7	24.3±10.7	21.8±9.6	26.4±10.9	22.8±10.2	p<0.01
Day 3-4-5	25.9±11.0	29.5±11.6	28.6±11.3	30.4±13.8	28.2±11.8	
**Contentedness**						
Day 1	27.2±11.0	25.5±11.0	24.4±11.4	24.9±11.8	26.6±10.6	p<0.05
Day 3-4-5	32.3±11.5	27.2±10.6	28.9±11.0	28.9±12.5	27.9±11.3	
**tVAS**						
Day 1	59.7±20.9	55.6±16.6	59.7±19.5	60.0±18.5	68.3±17.7	p<0.001
Day 3-4-5	80.5±13.3	75.8±16.2	79.8±13.5	76.2±13.0	80.4±13.4	

Abbreviations: POMS, Profile Of Mood States; KSS, Karolinska Sleepiness Scale; eVAS, emotional Visual Analogue Scale; B&L VAS, Bond & Lader Visual Analogue Scales; tVAS, thirst Visual Analogue Scale.

Significant time effects were present for fatigue-inertia (p<0.001), tension-anxiety (p<0.05) and vigor-activity (p<0.05) (POMS subscores), alertness (p<0.05) (Bond and Lader VAS subscore), sleepiness (p<0.001) (KSS) and emotional stability (p<0.01) (eVAS). The detailed time by time comparisons of all these variables (not shown) reported mood worsening at awakening (07:00) compared to the other time points.

No significant intervention by time interaction effects were present in HIGH.

### Effects of increased water intake in LOW drinkers

Significant intervention effects were found for the POMS scores (Table 3): the increased water intake resulted in a significant decrease in confusion-bewilderment (p = 0.05), fatigue-inertia (p<0.001) and vigor-activity (p<0.05). On the KSS, the LOW tended to show lower sleepiness scores (p = 0.07) after the switch in water intake. Another significant intervention effect was observed in the thirst scores, which detected a significant decrease in thirst (p<0.001). No other significant effect of increased water consumption was observed the LOW.

**Table 3:** Mean values and standard deviation for the different mood scales according to time point & study period (baseline vs mean of Day 3-4-5) for the low drinkers group consuming 1 L/day at baseline and 2.5 L/day during intervention days.

Outcome parameters	Time point					p value (for main intervention effect)
	07:00	14:00	16:00	18:00	20:00	
**POMS**						
**Tension**						
Day 1	−0.3±4.1	−1.9±1.9	−2.3±1.6	−1.6±1.8	−1.8±1.5	ns
Day 3-4-5	−1.3±1.4	−1.8±1.6	−1.7±1.6	−1.7±1.7	−1.8±1.7	
**Depression**						
Day 1	1.2±1.7	0.8±1.4	0.5±1.0	0.2±0.6	0.5±1.2	ns
Day 3-4-5	0.5±0.9	0.6±1.0	0.5±1.1	0.5±1.1	0.7±1.7	
**Anger**						
Day 1	1.8±2.8	1.1±1.5	0.5±0.8	0.4±0.6	0.5±1.0	ns
Day 3-4-5	0.9±1.6	0.6±1.1	0.4±0.9	0.7±1.4	0.6±1.1	
**Fatigue**						
Day 1	5.0±3.7	3.0±2.4	2.1±2.9	1.8±2.3	2.7±3.2	p<0.001
Day 3-4-5	2.3±2.3	1.8±1.8	1.9±1.9	1.7±1.7	1.8±1.8	
**Confusion**						
Day 1	0.8±4.0	−0.8±2.1	−1.0±2.2	−1.0±2.1	−0.5±2.0	p = 0.05
Day 3-4-5	−0.4±1.6	−1.2±1.8	−1.2±1.9	−1.1±1.7	−1.0±2.1	
**Vigor**						
Day 1	11.6±6.2	11.7±5.7	11.7±6.0	11.1±6.3	10.1±6.9	p<0.05
Day 3-4-5	6.7±5.2	10.2±6.2	10.8±6.3	10.4±6.2	9.7±6.4	
**KSS**						
Day 1	4.3±1.8	3.2±1.3	3.0±1.8	3.2±1.5	3.2±1.7	p = 0.0729
Day 3-4-5	4.6±1.4	2.8±1.4	2.7±1.2	2.5±1.1	2.7±1.1	
**eVAS**						
Day 1	68.7±14.9	71.0±13.2	68.1±16.1	72.6±13.5	70.2±16.4	ns
Day 3-4-5	61.1±12.0	71.5±12.9	74.3±14.4	75.6±14.2	75.5±13.6	
**B&L VAS**						
**Alertness**						
Day 1	37.0±17.5	30.4±14.8	30.5±16.8	28.4±15.0	32.9±16.6	ns
Day 3-4-5	41.6±16.7	28.4±15.0	27.2±15.6	26.7±13.9	28.3±15.3	
**Calmness**						
Day 1	26.8±20.1	23.4±15.1	25.0±15.2	26.1±13.4	24.7±13.7	ns
Day 3-4-5	24.1±23.7	25.3±25.2	24.8±22.0	26.5±22.3	24.4±21.7	
**Contentedness**						
Day 1	26.9±17.0	24.5±12.1	22.2±10.6	23.2±11.7	24.4±13.1	ns
Day 3-4-5	28.8±12.9	23.7±14.4	22.2±13.7	22.1±12.4	22.5±12.6	
**tVAS**						
Day 1	43.7±23.7	43.1±20.4	53.8±22.5	38.8±21.7	49.4±21.3	p<0.001
Day 3-4-5	55.8±20.4	31.8±18.7	34.4±19.6	30.0±20.7	33.5±20.5	

Abbreviations: POMS, Profile Of Mood States; KSS, Karolinska Sleepiness Scale; eVAS, emotional Visual Analogue Scale; B&L VAS, Bond & Lader Visual Analogue Scales; tVAS, thirst Visual Analogue Scale.

Significant time effects were found for several POMS scores (anger-hostility, p<0.01; confusion-bewilderment, p<0.01; fatigue-inertia, p<0.001 and tension-anxiety, p<0.05), for the KSS sleepiness score (p<0.001), the eVAS score (p<0.01), for the Bond and Lader alertness score (p<0.001) and for the thirst score (p<0.001). Detailed time analyses (time by time comparisons not shown) found worse mood at awakening (07:00) compared to the other time points.

Intervention by time interaction effects were all non-significant, except for the thirst scores. These analyses showed that the LOW indicated significantly higher thirst scores at awakening (07:00) after the water intake switch than at baseline (p<0.05). At 14:00, 16:00 and 20:00 thirst scores were lower after fluid increase than at baseline (p<0.05, p<0.001 and p<0.01 respectively).

## Discussion

This intervention study demonstrated that a daily water intake increase led to a significant mood improvement in habitual low drinkers, who reported less fatigue, less confusion, less thirst, and who tended to be less sleepy. Conversely, habitual high drinkers forced to reduce their daily water intake indicated that the restricted water intake negatively impacted their mood state; they indicated being more thirsty, less calm, less content, less vigorous, and reported lower positive emotions. Thus, the present research results suggest that an increase or decrease in habitual water intake have, respectively, an improving or worsening effect on mood and sensations depending upon an individual's habitual drinking habits (HIGH *vs.* LOW).

According to the few published studies addressing water consumption and mood, the hypothesis was that a reduction in water intake in HIGH would have the same effect as fluid deprivation [11]; that is having a detrimental effect primarily on sleep/wake parameters. Nevertheless, this hypothesis was not confirmed by our findings; the change in fluid intake induced a mood lowering as expected, but the effects were seen on nonspecific aspects of mood (calmness, contentedness and positive emotions) rather than on specific sleep/wake parameters. The reduced water intake in this study led to a significant decrease in vigor and had no effects on alertness and sleepiness feelings. The effects on the sleep/wake parameters were smaller than these observed in previous fluid deprivation protocols [9]–[11]. A comparison with the fluid deprivation protocols however needs to be made with caution, since this study did not aim to dehydrate the subjects. Instead, the reduced water intake in HIGH in the current study put the subjects into a situation of suboptimal hydration rather than a dehydration state. This situation of reduced water intake, instead of fluid deprivation, more closely mimics situations that can occur in daily life.

One surprising finding was that HIGH were less fatigued during the intervention period of reduced water intake which is not only contradictory to the research hypothesis, but also to the observed decrease in the vigor. One explanation for this observation is that the lower fatigue scores were not directly related to the change in water intake habits but rather to the change in life habits required during the study. During the inpatient period all volunteers refrained from physical activity, performed limited intellectual activities without any form of stress and were required to sleep eight hours per night. Consequently it seems plausible that they felt less fatigued after several inpatient days compared to in everyday life where they are exposed to the stressors and rhythms of daily life (e.g. work).

The LOW who increased their daily water intake reported less fatigue and less confusion. The water intake increase also tended to decrease sleepiness. This positive effect on alertness of water intake has previously been described in other experimental conditions [12], [13]. The water intake effect on happiness ratings of young adults in this study is in line with the results obtained in schoolchildren [18]. A contradictory observation was the significant decrease in vigor after the increase in water intake. However, a more detailed analysis showed that vigor scores were only different between experimental conditions at one time point, i.e. at awakening (07:00). Furthermore, the time effect analyses showed that mood lowering was observed in both groups at this same time point. Consequently, this decrease in vigor was not considered to be related to the water intake switch. Indeed, this mood lowering observed at awakening in most of the mood parameters was probably due to environmental/experimental conditions rather than to a change in water intake. It is likely that at baseline, the volunteers were motivated and curious, thus their mood was quite good. After three to six study days they were possibly less motivated to wake up in the morning for a long and uneventful study day.

Group comparison analyses showed that at baseline, mood in LOW and HIGH was similar except on subjective thirst feeling which was higher in HIGH than in LOW, and on the depression ratings which were also higher in HIGH. Both these group differences could be due to the fact that water intake was standardized (i.e.; given quantities at given time points) and was not an *ad libitum*. We hypothesize that the required water drinking program could have had a negative impact on thirst and depression ratings at baseline in the HIGH.

### Study limitations

Water intake standardization, defined as providing specified quantities of water at defined time points, may represent a bias in this study as indicated by the significant group differences in thirst and depression observed at baseline. In future research the effect of the structured drinking program should be taken into consideration when interpreting the results, at least on effects observed during the baseline period, as it seems to have an adverse effect on HIGH. It should also be noted that the drinking program during the intervention period was somewhat artificial because in real-life conditions, timing and quantities of drinking vary according to individuals and to the nature of fluids consumed [19], [29]. Indeed, in this study, the only fluid permitted during the intervention was water, and it is possible that replacing participants' habitual fluid intake with water only may impact some of the mood parameters studied. This possible confound was minimized to the extent possible by including only participants who did not habitually consume high quantities of caffeine or alcohol. Moreover, the study conditions may also have induced some biases in the results. As discussed previously, the several in-patient days with limited physical and intellectual activities in a young working population may have had a negative effect on mood ratings. A control group admitted for the same number of days but without a water intake intervention would have been an option to control or verify the in-patient effects on mood. Nevertheless, the fact that differential results were observed between LOW and HIGH suggests a true effect of the water intake intervention, as both groups experienced identical inpatient study conditions but reported different mood changes. A final limitation to acknowledge is the fact that HIGH included only females. This may have affected the results as it was demonstrated that dehydration may impact males and females differently [6]. Indeed, this previous research demonstrated than females were more sensitive to the effects of mild dehydration than men. Consequently, the results on the impact of a reduced water intake on mood cannot be generalized.

## Conclusion

This study addresses the effects of a change in water intake on mood and physiological sensations in adults. The results showed that a switch toward an increase in water intake has especially beneficial effects on sleep/wake moods of habitual low-volume drinkers. The switch toward a decrease in water intake has detrimental effects on mood rating of habitual high-volume drinkers, including reduced feelings of calmness, satisfaction and positive emotions. Further research is necessary to confirm these results, using a less restrictive drinking program and a more appealing daily schedule of activities so that the effects of water intake changes can be more accurately assessed.
